# Public Health in the United States—IV

**Published:** 1906-02-03

**Authors:** 


### PUBLIC HEALTH IN THE UNITED STATES—IV.

(*Continued from page* 295.)

### Food other than Dairy Products.

There are great differences in food laws and in their administration. The control of food substances as regards both legislation and execution is sometimes national, sometimes State, and sometimes municipal. The federal Government, by virtue of its authority to control interstate and foreign commerce, has legislated concerning the wholesomeness and purity of food supplies and has a large force of inspectors constantly at work. The chief field covered by the federal officers is the inspection of cattle intended for slaughter, and also of the various products sent out from the slaughter-houses. Much of the legislation, especially that concerning adulterations, is State legislation, and the execution of these laws is vested in State officers. The execution of milk laws is more often in the hands of local boards of health; as regards the use of food from diseased animals, State laws are common and State inspection also. Local regulations are, of course, found on all of these subjects, but the special field of local legislation is the control of the sale of foods which are undergoing putrefactive changes. Putrefaction in this connection, of course, is not confined to its popular use, but embraces any of those degenerative changes which are produced by the action of saprophytic organisms, though the materials may not have become actually rotten.

### Unwholesome Food.

Some of the earliest legislation in regard to food was of a very general character, and similar laws are to-day found on the Statute books of most of the States. Food from animals which are diseased at the time of killing is assumed to be unwholesome. Animals become feverish from driving or transportation, and many cities forbid their being slaughtered under such conditions. Indiana and a number of cities forbid the use of maimed or wounded animals as food. In Atlanta and Cincinnati it is forbidden to use the flesh of pregnant animals as food. It is very common to forbid the sale of meat of animals that have died from disease or accident. In New York and many other places "no meat or dead animal above the size of a rabbit shall be taken to any public or private market for food, until the same shall have fully cooled after killing, nor until the entrails, heads and feet (except of poultry and game, and except the head and feet of swine) shall have been removed." The question as to whether poultry should be sold without having the viscera removed has been much discussed. If the entrails remain the odours and taste from the offensive matter are absorbed by the surrounding tissues which are therefore more or less disagreeable to the consumer. On the other hand, it is claimed by the dealers decomposition of the muscular tissues takes place sooner when the viscera are removed. The consumer then would naturally prefer that the poultry be promptly drawn, while the dealers prefer that they should not be, as they thus keep longer in a saleable condition. The Massachusetts law requires that they should be drawn. In Minnesota plucked poultry and game and rabbits must be drawn. In Detroit the crops of poultry must be removed before they are sold. The practice of blowing carcasses—*i.e.*, injecting air under the superficial fascia—is resorted to to a considerable extent in order that the meat shall present a better appearance, as the air in the tissues makes it look like fat. It is a reprehensible practice as it is in the nature of a fraud, and, furthermore, it is not desirable to introduce saliva from a butcher into the animal through a dirty pipe stem, an accident which doubtless sometimes occurs. Hence in a number of cities the sale of meat so treated is forbidden. In New Orleans it is provided that the carcasses shall only be blown by means of a bellows, and that no animals over fifteen months of age shall be blown at all.

### Adulteration and Substitution.

Laws in regard to adulteration are found in both federal and State statutes and in municipal ordinances. Of these by far the most important are the State laws. The only federal statute in regard to adulterations forbids the importation of adulterated foods, drugs, or liquors. The adulteration of drugs is specifically forbidden in seventeen States, but usually the term is used in connection with "food, liquors," etc., in a general law. Vinegar is especially mentioned in nearly a dozen States as among the substances not to be adulterated. The chief adulterant of molasses, sugar, honey, etc., is glucose, and its use for this purpose is forbidden by a statute which is found in New York, Colorado, and several other States. The law forbids anyone to mix glucose with syrup or sugar in Iowa, and with syrup, honey, or sugar in other States; but in each of the States it is permissible so to use glucose provided the percentage of glucose is marked on the package and made known to the buyer. In Massachusetts, syrup and molasses must bear the maker's name. The question of the injurious effects of alum in baking-powders has been much discussed. Opinions, however, differ as to whether the small amount of alum remaining in food in which alum baking-powder is used is injurious, and consequently opinions differ as to the advisability of legislation on the subject. In most States there is no prohibition of such a use of alum, but in Minnesota there was a stringent law to forbid the sale of baking-powders containing alum unless that fact was plainly marked upon the package. This was afterwards repealed, and the present law, which is very explicit, requires that all baking-powders shall have the ingredients and amounts marked on the package. In some States there are laws and rules relating to poisonous colouring-matter in children's toys and confectionery. The San Francisco Board of Health has established a standard of tomato catsup. It must contain 25 per cent. of organic solids, including ash, and be free from antiseptics and colouring matter. The practice of adding beef-suet and cotton-seed oil to lard met with great opposition among the manufacturers of pure lard, and as a result of the agitation laws against the adulteration of lard are found in a number of States. The most stringent and complete law is that of Minnesota. This law forbids the sale "as lard of any substance not the legitimate and exclusive product of the fat of the hog" unless it shall be labelled in letters not less than one inch in length, and shall have the name of the manufacturer and the location of the manufactory in letters of the same size, and the names and proportions of the ingredients in letters one half-inch in length. If it is a "mixture or compound of animal or vegetable oils or fats other than hog-fat in the form of lard," it shall be labelled "Lard Substitutes." If it is a mixture of the above with lard it shall be labelled "Adulterated lard." Dealers and traders must also mark goods in a similar manner with letters half the above size and must give a card to the customer stating the facts. The penalties attaching to the violation of the food laws, particularly the adulteration laws, are the most severe found in any of the so-called sanitary legislation. The injury to health is slight, but the penalty is great, while for the violation of laws which may produce vast injury to health the penalty is often nominal. The severity of the food-law penalties does not represent the feelings of the public in regard to the importance of the subject, but rather the legislative influence of the commercial interests which usually secure this class of legislation. Violation of food laws are, of course, punished by fine or imprisonment. The fines range from $10 minimum in Tennessee to a $2,000 maximum in Illinois, and the imprisonment from thirty days to five years. $300 to $500 or one year are common maxima.

### Dairy Products.

The adulteration of butter and cheese or the sale of imitations very seriously affect the pockets of the dairymen. As dairymen form a large and influential class throughout the country, it might be expected that stringent legislation concerning this class of adulteration would be very common. As a matter of fact, laws relating to butter and cheese are found in all but six of the States and territories. Most of the agricultural States, and also the district of Columbia, have statutes forbidding the manufacture and sale of adulterated cheese. Sophisticated cheese is not so common as artificial butter, but its manufacture and sale is opposed by the same interests, and in many States it is treated of in the same statute with butter. A few States have laws to secure the delivery of pure milk or cream to creameries. There is, however, a great mass of legislation directed against the manufacture and use of butter substitutes. It is possible to so treat partly rancid butter as to make a product which, while of poor grade, is yet saleable. Several States have laws against selling such butter without marking it as such.

### Milk.

Milk is in far more urgent need of protection than any other food. It is far more liable to adulteration and pollution than any other important food, and its adulteration and pollution are attended with far more serious consequences. Cows' milk is the sole food of a large number of infants, the chief food of nearly all young children, and the mainstay in the nourishment of adults in sickness and convalescence. The importance of protecting this food has long been recognised, and far greater efforts have been made for this than for the control of any other food material; but the ease with which milk can be adulterated, and the great profit of the operation, are obstacles which can be overcome only by the utmost vigilance in the enforcement of stringent laws; and the labour and care which are required on the part of the producer to secure a clean milk are so great that little progress has yet been made towards obtaining this much to be desired result. The temptations for milk adulterating are so great that many yield among the producers, and also among the middlemen and peddlers, when such intervene between the producer and consumer. The subject naturally divides itself into two parts : First, the prevention of the sale of adulterated or diluted milk, or milk poor in nutritive ingredients; and, secondly, the attempt to secure clean milk—milk which is free from dirt, and particularly milk which is free from the micro-organisms, either pathogenic germs or those which simply injure the quality of the milk. The first of these has received by far the greater attention, and for thirty years an active warfare has been waged in the larger cities against diluted milk. The cleanliness of milk has, however, only lately received much attention, and, in fact, it is only lately that the true importance of cleanliness has been understood. There is no federal milk law, but much has been done in the spheres of both State and municipal legislation. As in all food legislation, the State laws are the most important, though many valuable municipal regulations are found, particularly concerning cows and stables. The use of colouring matter in milk has been forbidden in many States, as well as the use of preservatives. Most milk laws forbid the sale of milk from sick or diseased cows. The stalling of cows has a marked effect upon the milk, hence the provision of the New York law that milk shall be considered "adulterated when drawn from cows kept in a crowded or unhealthy condition." The temptation to sell as pure milk milk from which a part of the cream has been removed is almost as great as is the temptation to sell watered milk. The sale of such milk, even as skimmed milk, is therefore prohibited in many States, along with milk that is "impure, adulterated, or unwholesome." The sale of such milk is absolutely prohibited in Georgia, Indiana, Kansas, Kentucky, Nebraska, Vermont, Virginia, and Washington. In Michigan it is forbidden to sell it to creameries, and in New York it must not be sold except to skim cheese factories, except when it is properly designated, and its sale is absolutely prohibited in the city of New York. Several of the above-mentioned States are engaged very extensively in the manufacturing of cheese and butter, and it is to protect the creameries chiefly that these provisions are made. Massachusetts, New York, and Ohio are the only States which have regulations concerning condensed milk. The Massachusetts statute merely requires that every package of condensed milk shall be marked with the name of the maker, the brand, and the contents. In most cities a person may not sell milk without a license, and in such cases the revocation of the license after violation of the milk laws is a most severe and effectual means of punishment. One of the most valuable means of controlling the milk supply is by licensing the dealers in that commodity. This is a very general practice in American cities. The recent movement of health officers to secure good milk requires the control, or at least inspection of the conditions under which the milk is produced, and hence a knowledge of the producers is required. Within the last few years there has grown up a very strong desire on the part of sanitary officials and progressive medical men for clean milk. At present this desire only affects a small part of the consumers, the great body of whom are content to use the dirty milk which is generally furnished by dairymen. There is a very great danger of gastro-intestinal disturbances in infants and young children from the ingestion of milk containing large numbers of bacteria. In order to obtain clean milk for the public it is necessary to control the methods of producing milk and handling it on the way from the producer to the customer. As soon as the danger of giving to infants milk rich in bacteria was recognised, the attempt was made to overcome the difficulty by sterilisation; but it was soon found that this rendered the milk less digestible, and that nearly as good practical results could be obtained by a partial sterilisation at lower temperature, by which all but the most resistent germs would be destroyed, and at which temperature the milk would be only slightly altered. This pasteurisation, like sterilisation, was first done in private families, on the advice of physicians, but soon certain dairymen took up the practice, in order to meet the demand of the better class of their customers, who called for it on their physicians' advice. At present there are in most large cities private dealers or dairymen who supply a limited demand for pasteurised milk. The advantages of pasteurised milk as an infants' food are so great, and the chances of the poor, who need it most, appreciating it and paying for it are so slight, that the necessity for its distribution quickly appealed to the hearts of the charitable. The distribution of pasteurised milk at a price below cost has become a feature of the charitable work in New York, Brooklyn, Cleveland, Rochester, and other cities. The most important of these charities is that conducted by the Hon. Nathan Strauss, of New York, and begun by him in 1893. Through the efforts of Mr. Strauss, 596,677 bottles of pasteurised milk were distributed in 1900. During the years that his milk has been coming into use there has been a decided falling-off in infant mortality in New York, and it is claimed that it is due to that cause.

